# 3,3′-(2,2′-Bi-1*H*-imidazole-1,1′-diyl)dipropanol

**DOI:** 10.1107/S1600536808043377

**Published:** 2009-01-08

**Authors:** Tao Zhang, Hong-Ze Liang

**Affiliations:** a State Key Laboratory Base of Novel Functional Materials and Preparation Science, Institute of Solid Materials Chemistry, Faculty of Materials Science and Chemical Engineering, Ningbo University, Ningbo 315211, People’s Republic of China

## Abstract

In the title compound, C_12_H_18_N_4_O_2_, unlike other unconjugated disubstituted biimidazole derivatives reported so far, the two imidazole rings in a *trans* conformation exhibit a large planar rotation angle of 51.27 (4)°, and consist of half-mol­ecule asymmetric units related by a twofold rotation. The mol­ecules are linked into a three-dimensional framework with a parallel laminated construction *via* O—H⋯N and C—H⋯O inter­actions.

## Related literature

For background to 2,2′-biimidazole derivatives, see: Forster *et al.* (2004 ▶); Fortin & Beauchamp (2000 ▶); Fu *et al.* (2007 ▶); Ion *et al.* (2007 ▶); Mao *et al.* (2003 ▶); Pereira *et al.* (2006 ▶); Xiao & Shreeve (2005 ▶); Xiao *et al.* (2004 ▶). For other unconjugated 1,1′-disubstituted compounds, see: Barnett *et al.* (1997 ▶, 2002 ▶); Secondo *et al.* (1996 ▶, 1997 ▶). For the synthesis, see: Barnett *et al.* (1999 ▶)
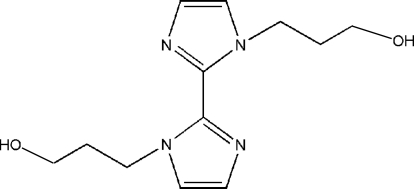


## Experimental

### 

#### Crystal data

C_12_H_18_N_4_O_2_
*M* *_r_* = 250.30
Monoclinic, 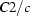

*a* = 15.812 (3) Å
*b* = 9.5961 (19) Å
*c* = 9.3194 (19) Å
β = 119.44 (3)°
*V* = 1231.5 (6) Å^3^
*Z* = 4
Mo *K*α radiation
μ = 0.10 mm^−1^
*T* = 295 (2) K
0.56 × 0.48 × 0.37 mm

#### Data collection

Rigaku R-AXIS RAPID diffractometer
Absorption correction: multi-scan (*ABSCOR*; Higashi, 1995 ▶) *T*_min_ = 0.948, *T*_max_ = 0.970
4715 measured reflections
1400 independent reflections
1183 reflections with *I* > 2σ(*I*)
*R*_int_ = 0.015

#### Refinement

*R*[*F* ^2^ > 2σ(*F* ^2^)] = 0.036
*wR*(*F* ^2^) = 0.104
*S* = 1.07
1400 reflections
86 parameters
H atoms treated by a mixture of independent and constrained refinement
Δρ_max_ = 0.17 e Å^−3^
Δρ_min_ = −0.23 e Å^−3^

### 

Data collection: *RAPID-AUTO* (Rigaku, 1998 ▶); cell refinement: *RAPID-AUTO*; data reduction: *CrystalStructure* (Rigaku/MSC, 2002 ▶); program(s) used to solve structure: *SHELXS97* (Sheldrick, 2008 ▶); program(s) used to refine structure: *SHELXL97* (Sheldrick, 2008 ▶); molecular graphics: *SHELXTL* (Sheldrick, 2008 ▶); software used to prepare material for publication: *SHELXTL*. 

## Supplementary Material

Crystal structure: contains datablocks global, I. DOI: 10.1107/S1600536808043377/at2693sup1.cif

Structure factors: contains datablocks I. DOI: 10.1107/S1600536808043377/at2693Isup2.hkl

Additional supplementary materials:  crystallographic information; 3D view; checkCIF report

## Figures and Tables

**Table 1:** Hydrogen-bond geometry (Å, °)

*D*—H⋯*A*	*D*—H	H⋯*A*	*D*⋯*A*	*D*—H⋯*A*
O1—H9⋯N2^i^	0.89 (2)	1.92 (2)	2.8047 (17)	175
C2—H2⋯O1^ii^	0.93	2.59	3.5019 (17)	166

Symmetry codes: (i) 
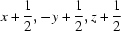
; (ii) 
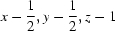
.

**Fig. 1.**
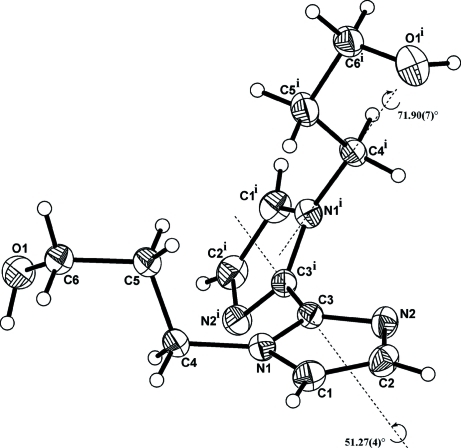
A perspective view of (I) with the atom-labelling scheme, showing that the two imidazole rings are distinctly non-coplanar. Displacement ellipsoids are drawn at the 40% probability level and H atoms are shown as small spheres of arbitrary radius.

**Fig. 2.**
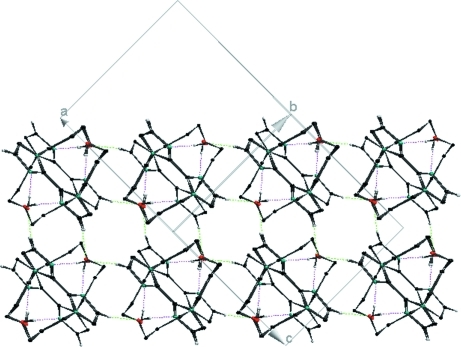
A perspective view of the three–dimensional framework with parallellaminated construction containing two sheets from [011] direction, showing the packing mode and the interactions of hydrogen bonds O–H···N (pink dashed lines in the electronic version of the paper) and C–H···O (green dashed lines in the electronic version). All H atoms not involved in the hydrogen-bond motifs have been omitted for clarity.

## supplementary crystallographic information

### Comment

2,2'-Biimidazole (H_2_biim) derivatives as versatile ligands are widely used in the construction of metal complexes (Ion *et al.*, 2007; Pereira *et al.*, 2006; Fortin *et al.*, 2000). In addition to the study of metal complexes, attention has also been devoted to the polymeric systems (Fu *et al.*, 2007; Forster *et al.*, 2004; Mao *et al.*, 2003) and the ionic liquids (Xiao *et al.*, 2005, 2004). The title compound (I), as a hydroxy-terminated derivative can be easily synthesized by Michael addition of dianion biim^2-^ to alkyl acrylate (Barnett *et al.*, 1999) and subsequent mild reduction by NaBH_4_ in an excellent yield.

The crystal structure of (I), shown in Fig. 1, adopts a *trans* conformation, and consists of half-molecule asymmetric units related by a twofold rotation. Exceptionally, unlike the other unconjugated 1,1'-disubstituted compounds reported before (Barnett *et al.*, 1997, 2002; Secondo *et al.*, 1996, 1996), the imidazole rings of the investigated compound (I) exhibit a rather large torsion angle of 51.27 (4)°, as shown in Fig. 1. The terminal hydroxyl groups are responsible for this noncoplanarity. The imdazole rings are forced to rotate by intermolecular interactions *via* strong O–H···N hydrogen bonds. The N1–C4–C5–C6 moiety is essentially planar with an r.m.s. deviation of 0.054 Å. This plane is at angle of 71.90 (7)° with respect to the adjacent imidazole ring, which is consistent with the reported analogous compounds referenced before. While the atom O1 does not lie in the N1–C4–C5–C6 plane, rather it lies 1.116 Å from this plane. The bond length and bond angle in (I) are within normal ranges.

The molecules of (I) are linked into a three-dimensional framework with parallel laminated construction by a combination of one strong O–H···N hydrogen bond and the other weak C–H···O hydrogen bond (Table 1). It can be analyzed in terms of thousands of one-dimensional substructures, linked by C–H···O hydrogen bonds, which generate series of two-dimensional sheets through C–H···O hydrogen bonds (Fig.2).

### Experimental

1,1'-Di(ethylpropionato)-2,2'-biimidazole (0.80 g, 2.40 mmol) was dissolved in absolute ethyl alcohol (50 ml). To this mixture was added batchly NaBH_4_ (0.906 g, 24.0 mmol). The mixture was heated to reflux for 3 h. Evaporating the solvent and added to 30 ml H_2_O yield a transparent solution. Adjusting the PH of the solution to 8–9 with 1*M* HCl led to a white suspension. The mixture was filtered, then the filtrate extracted with chloroform (10 ml) three times. The combined organic layer was washed with H_2_O (5 ml) two times, dried by MgSO_4_. After evaporating the solvent, the residue was chromatographed on silica gel. Elution with CH_3_CO_2_C_2_H_5_/C_2_H_5_OH (10:1) afforded a white solid (0.52 g, 86.7%). Tetragonal crystals of the titled compound were formed by evaporating saturated petroleum ether/chloroform solution slowly.

Similarly, the title compound(I) can be synthesized by reduction of other 1,1'-di(alkylpropionato)-2,2??-biimidazole (alkyl = methyl, butyl) which was prepared according to the published procedure (Barnett *et al.*, 1999) with NaBH_4_ in anhydrous ethyl alcohol or with LiAlH_4_ in anhydrous ethereal solution. The yields ranges from 44 to 85°.

### Refinement

All H atoms were placed in geometrically idealized positions and constrained to ride on their parent atoms (C—H = 0.93 and = 0.97 Å; O—H = 0.82 Å) and *U*_iso_(H) values were taken to be equal to 1.2 *U*_eq_(C) and 1.5U_eq_(O).

### Crystal data

**Table:**

C_12_H_18_N_4_O_2_	*F*(000) = 536
*M**_r_* = 250.30	*D*_x_ = 1.350 Mg m^−^^3^
Monoclinic, *C*2/*c*	Mo *K*α radiation, λ = 0.71073 Å
Hall symbol: -C 2yc	Cell parameters from 1400 reflections
*a* = 15.812 (3) Å	θ = 3.1–27.5°
*b* = 9.5961 (19) Å	µ = 0.10 mm^−^^1^
*c* = 9.3194 (19) Å	*T* = 295 K
β = 119.44 (3)°	Block, colourless
*V* = 1231.5 (6) Å^3^	0.56 × 0.48 × 0.37 mm
*Z* = 4	

### Data collection

**Table:**

Rigaku R-AXIS RAPID diffractometer	1400 independent reflections
Radiation source: fine-focus sealed tube	1183 reflections with *I* > 2σ(*I*)
graphite	*R*_int_ = 0.015
ω scans	θ_max_ = 27.5°, θ_min_ = 3.1°
Absorption correction: multi-scan (*ABSCOR*; Higashi, 1995)	*h* = −20→20
*T*_min_ = 0.948, *T*_max_ = 0.970	*k* = −12→12
4715 measured reflections	*l* = −12→12

### Refinement

**Table:**

Refinement on *F*^2^	Primary atom site location: structure-invariant direct methods
Least-squares matrix: full	Secondary atom site location: difference Fourier map
*R*[*F*^2^ > 2σ(*F*^2^)] = 0.036	Hydrogen site location: inferred from neighbouring sites
*wR*(*F*^2^) = 0.104	H atoms treated by a mixture of independent and constrained refinement
*S* = 1.07	*w* = 1/[σ^2^(*F*_o_^2^) + (0.0631*P*)^2^ + 0.3657*P*] where *P* = (*F*_o_^2^ + 2*F*_c_^2^)/3
1400 reflections	(Δ/σ)_max_ < 0.001
86 parameters	Δρ_max_ = 0.17 e Å^−^^3^
0 restraints	Δρ_min_ = −0.23 e Å^−^^3^

### Special details

**Table:**

Geometry. All e.s.d.'s (except the e.s.d. in the dihedral angle between two l.s. planes) are estimated using the full covariance matrix. The cell e.s.d.'s are taken into account individually in the estimation of e.s.d.'s in distances, angles and torsion angles; correlations between e.s.d.'s in cell parameters are only used when they are defined by crystal symmetry. An approximate (isotropic) treatment of cell e.s.d.'s is used for estimating e.s.d.'s involving l.s. planes.
Refinement. Refinement of *F*^2^ against ALL reflections. The weighted *R*-factor *wR* and goodness of fit *S* are based on *F*^2^, conventional *R*-factors *R* are based on *F*, with *F* set to zero for negative *F*^2^. The threshold expression of *F*^2^ > σ(*F*^2^) is used only for calculating *R*-factors(gt) *etc*. and is not relevant to the choice of reflections for refinement. *R*-factors based on *F*^2^ are statistically about twice as large as those based on *F*, and *R*- factors based on ALL data will be even larger.

### Fractional atomic coordinates and isotropic or equivalent isotropic displacement parameters (Å^2^)

**Table:**

	*x*	*y*	*z*	*U*_iso_*/*U*_eq_	
C1	0.03257 (9)	0.22372 (12)	0.47224 (14)	0.0366 (3)	
H1	0.0646	0.2434	0.4134	0.044*	
C2	−0.05565 (9)	0.16313 (12)	0.41318 (14)	0.0375 (3)	
H2	−0.0946	0.1336	0.3050	0.045*	
C3	−0.00459 (7)	0.20468 (10)	0.66867 (13)	0.0280 (3)	
C4	0.15367 (7)	0.32897 (11)	0.74530 (14)	0.0328 (3)	
H3	0.1970	0.3292	0.6993	0.039*	
H4	0.1869	0.2829	0.8516	0.039*	
C5	0.13141 (8)	0.47728 (12)	0.76898 (17)	0.0418 (3)	
H5	0.0930	0.4771	0.8241	0.050*	
H6	0.0930	0.5207	0.6620	0.050*	
C6	0.22260 (8)	0.56240 (12)	0.86937 (16)	0.0390 (3)	
H7	0.2635	0.5562	0.8189	0.047*	
H8	0.2048	0.6594	0.8674	0.047*	
N1	0.06543 (6)	0.25027 (9)	0.63558 (11)	0.0300 (2)	
N2	−0.07917 (6)	0.15167 (10)	0.53523 (11)	0.0340 (3)	
O1	0.27596 (7)	0.51756 (11)	1.03437 (12)	0.0475 (3)	
H9	0.3207 (14)	0.460 (2)	1.037 (2)	0.073 (6)*	

### Atomic displacement parameters (Å^2^)

**Table:**

	*U*^11^	*U*^22^	*U*^33^	*U*^12^	*U*^13^	*U*^23^
C1	0.0423 (6)	0.0386 (6)	0.0319 (6)	0.0015 (5)	0.0206 (5)	0.0000 (4)
C2	0.0425 (6)	0.0373 (6)	0.0258 (6)	−0.0003 (4)	0.0114 (4)	−0.0030 (4)
C3	0.0253 (5)	0.0265 (5)	0.0287 (6)	0.0014 (4)	0.0107 (4)	0.0005 (4)
C4	0.0246 (5)	0.0324 (5)	0.0387 (6)	−0.0004 (4)	0.0134 (4)	−0.0013 (4)
C5	0.0285 (6)	0.0333 (6)	0.0525 (8)	0.0020 (4)	0.0114 (5)	−0.0050 (5)
C6	0.0330 (6)	0.0316 (5)	0.0477 (7)	−0.0014 (4)	0.0163 (5)	−0.0033 (5)
N1	0.0284 (5)	0.0313 (5)	0.0295 (5)	0.0000 (3)	0.0136 (4)	−0.0014 (3)
N2	0.0318 (5)	0.0351 (5)	0.0281 (5)	−0.0035 (4)	0.0093 (4)	−0.0020 (3)
O1	0.0407 (5)	0.0592 (6)	0.0391 (6)	0.0028 (4)	0.0168 (4)	−0.0102 (4)

### Geometric parameters (Å, °)

**Table:**

C1—C2	1.3536 (18)	C4—H3	0.9700
C1—N1	1.3692 (16)	C4—H4	0.9700
C1—H1	0.9300	C5—C6	1.5151 (16)
C2—N2	1.3638 (16)	C5—H5	0.9700
C2—H2	0.9300	C5—H6	0.9700
C3—N2	1.3245 (14)	C6—O1	1.4093 (17)
C3—N1	1.3595 (14)	C6—H7	0.9700
C3—C3^i^	1.450 (2)	C6—H8	0.9700
C4—N1	1.4697 (14)	O1—H9	0.89 (2)
C4—C5	1.5081 (16)		
			
C2—C1—N1	106.51 (11)	C4—C5—H5	109.1
C2—C1—H1	126.7	C6—C5—H5	109.1
N1—C1—H1	126.7	C4—C5—H6	109.1
C1—C2—N2	110.15 (10)	C6—C5—H6	109.1
C1—C2—H2	124.9	H5—C5—H6	107.9
N2—C2—H2	124.9	O1—C6—C5	112.75 (11)
N2—C3—N1	111.05 (10)	O1—C6—H7	109.0
N2—C3—C3^i^	124.54 (11)	C5—C6—H7	109.0
N1—C3—C3^i^	124.26 (11)	O1—C6—H8	109.0
N1—C4—C5	112.13 (9)	C5—C6—H8	109.0
N1—C4—H3	109.2	H7—C6—H8	107.8
C5—C4—H3	109.2	C3—N1—C1	106.62 (10)
N1—C4—H4	109.2	C3—N1—C4	127.45 (10)
C5—C4—H4	109.2	C1—N1—C4	125.51 (10)
H3—C4—H4	107.9	C3—N2—C2	105.68 (10)
C4—C5—C6	112.29 (9)	C6—O1—H9	105.3 (12)

Symmetry codes: (i) −*x*, *y*, −*z*+3/2.

### Hydrogen-bond geometry (Å, °)

**Table:**

*D*—H···*A*	*D*—H	H···*A*	*D*···*A*	*D*—H···*A*
O1—H9···N2^ii^	0.89 (2)	1.92 (2)	2.8047 (17)	175
C2—H2···O1^iii^	0.93	2.59	3.5019 (17)	166

Symmetry codes: (ii) *x*+1/2, −*y*+1/2, *z*+1/2; (iii) *x*−1/2, *y*−1/2, *z*−1.
